# Serum uric acid level predicts the progression of amyotrophic lateral sclerosis following treatment with edaravone

**DOI:** 10.1080/13510002.2022.2051964

**Published:** 2022-03-16

**Authors:** Hee Jo Han, Ha Young Shin, Young-Chul Choi, Seung Min Kim, Seung Woo Kim

**Affiliations:** a Department of Neurology, Yonsei University College of Medicine, Seoul, South Korea; b Department of Neurology, Yongin Severance Hospital, Yonsei University Health System, Yongin, South Korea

**Keywords:** Uric acid, amyotrophic lateral sclerosis, ALS, edaravone, prognosis, oxidative stress, radical scavenger, motor neuron

## Abstract

**Introduction:**

Uric acid and edaravone might exert a neuroprotective effect in amyotrophic lateral sclerosis (ALS) by reducing oxidative stress. We analyzed whether the treatment effect of edaravone is pronounced in patients whose uric acid level increased after the treatment with edaravone.

**Materials and methods:**

Forty patients with ALS who underwent treatment with edaravone were included. Baseline uric acid level and the rate of decline in uric acid after edaravone treatment were recorded. The rate of change of ALS functional rating scale-revised (ΔALSFRS-R/month) was calculated based on baseline ALSFRS-R score and ALSFRS-R score 6–24 weeks after the treatment.

**Results:**

The serum uric acid levels decreased after treatment in 26 (65%) patients and increased in 12 (30%) patients. The ΔALSFRS-R/month was significantly faster in patients whose uric acid decreased (median 1.5 [Q1–Q3, 0.7–3.1]) than in patients whose uric acid increased (0.2 [0–1.0], *p* = 0.021). A high baseline uric acid level and low rate of decline in uric acid was associated with slower disease progression after adjusting for age, initial symptoms, and riluzole administration (*p* = 0.030 and *p* = 0.041, respectively).

**Discussion:**

High baseline values and low rate of decline in uric acid may predict slow disease progression in ALS patients treated with edaravone.

## Introduction

Amyotrophic lateral sclerosis (ALS) is a fatal neurodegenerative disease that affects the upper and lower motor neurons, leading to muscle weakness and wasting. Although the exact pathomechanism of ALS remains unclear, increased oxidative stress has been suggested as one of the potential causes of ALS. Previous studies have demonstrated that biomarkers for oxidative stress, including 3-nitrotyrosine, 8-hydroxy-2′-deoxyguanosine, and 4-hydroxynonenal, increase in patients with ALS [1]. Uric acid is a natural antioxidant in human plasma. Thus, there have been efforts to elucidate the beneficial role of uric acid in patients with ALS. Previous studies have demonstrated that serum uric acid levels are lower in patients with ALS than in healthy persons and tend to decrease as the disease progresses [2–5]. The studies may imply that increased oxidative stress in ALS decreases the level of uric acid. In addition, patients with ALS with high uric acid levels presented with slow disease progression [5–8]. These results suggest that uric acid may play a neuroprotective role in ALS by reducing oxidative stress.

Edaravone is a drug approved by the United States Food and Drug Administration in May 2017 to treat ALS [9]. Although the exact mechanism of action remains unclear, its main function is that of a free-radical scavenger. It is hypothesized that edaravone slows disease progression by protecting motor neurons from oxidative damage. Although both uric acid and edaravone function as free-radical scavengers, it is postulated that the antioxidative effect of edaravone is significantly higher than that of uric acid. A previous study demonstrated that the reactivity of edaravone with peroxynitrite, which is a strong oxidant, is nearly 30 times that of uric acid [10]. Decrease in anti-oxidative activity and increase in serum uric acid levels after the administration of edaravone were also demonstrated in previous studies [11, 12]. This could imply that edaravone and uric acid have a synergic effect in scavenging free radicals.

In the present study, we hypothesized that the patients whose uric acid level increased after edaravone treatment might have slower disease progression than the patients whose uric acid level decreased after edaravone treatment. To test this hypothesis, we dichotomized the patients based on the change in uric acid after edaravone treatment and compared the rate of decline of ALS functional rating scale-revised (ALSFRS-R) between the two groups.

## Materials and methods

### Patient enrollment

We retrospectively investigated the electronic medical records of patients diagnosed with ALS who underwent treatment with edaravone at two different hospitals (Severance Hospital and Gangnam Severance Hospital) between January 2017 and October 2020. The diagnosis of ALS was based on the revised El Escorial criteria, and those who fulfilled the criteria for ‘definite,’ ‘probable,’ ‘probable-laboratory-supported,’ or ‘possible’ ALS were selected [13]. All patients received edaravone following the recommended dosage. Edaravone was administered once per day for 14 consecutive days during the first treatment cycle followed by a two-week drug-free period. Subsequent cycle consisted of 10-day treatment followed by two-week drug cessation. We included patients who met the following criteria: 1) baseline serum uric acid level was measured within 7 days before the initiation of edaravone treatment; 2) follow-up serum uric acid level was measured within 14 days after the initiation of first edaravone treatment; 3) baseline ALSFRS-R score was assessed during admission for the first edaravone treatment cycle; and 4) follow-up ALSFRS-R score was assessed 6–24 weeks after the first edaravone treatment. The exclusion criteria were 1) presence of fatal diseases other than ALS that could affect functional status; 2) presence of other medical conditions that could affect the uric acid level, including history of stroke or myocardial infarction, renal dysfunction, acute infection, or acute inflammatory state; and 3) use of uric acid-lowering medications. To compare the uric acid levels between the patients with ALS and healthy controls, age-, sex-, and body mass index (BMI)-matched controls were selected from healthy individuals whose uric acid levels were measured during health screening at our institution during the study period. This research was approved by the University of Yonsei Institutional Review Board (Approval No. 4-2020-0198) and conducted in accordance with the Declaration of Helsinki. The ethics committee waived the need for informed consent for the study.

### Data collection and definition

Demographic data and clinical features including sex, age, weight, height, other medical conditions, disease duration before treatment, initial presentation (limb-onset, bulbar-onset, or respiratory-onset), disease severity, and serum uric acid level of the patients were recorded by reviewing their medical records. Concomitant administration of riluzole – another drug approved to treat ALS—was also assessed. Disease severity was evaluated using the Japanese ALS severity classification and ALSFRS-R score. Japanese ALS severity classification ranges from 1 (able to work or perform housework) to 5 (using a tracheostomy or tube feeding) [14]. ALSFRS-R is a validated rating instrument used to assess the functional status of patients with ALS. The ALSFRS-R score ranges from 0 to 48, with lower scores indicating worse functional state [15]. Functional outcome of the patients was defined as the rate of change of the ALSFRS-R score (ΔALSFRS-R/month). The rate of change of ALSFRS-R score was calculated by dividing the change in the ALSFRS score (ALSFRS-R score at baseline − ALSFRS-R score at follow-up) by the time interval between the initial and follow-up assessments.

### Uric acid assessment

Baseline serum uric acid levels were measured during hospitalization prior to edaravone treatment. Follow-up uric acid levels were measured after the initiation of the first cycle of edaravone treatment. When the serum uric acid level was measured ≥ 2 times during the first cycle of edaravone treatment, the result of the blood test closest to the 7th day after the initiation of treatment was used. The rate of decline in serum uric acid level was calculated by dividing the change in the uric acid level (baseline uric acid level – follow-up uric acid level) by the baseline uric acid level. In order to assess whether the change of uric acid level is persistently observed throughout different time points, we additionally analyzed the result of the blood test closest to the 14th day after the initiation of the first cycle of edaravone treatment. We also analyzed the uric acid levels before and after the treatment in the patients who underwent their second administration of edaravone treatment in an outpatient setting.

### Statistical analysis

The data are expressed as mean ± standard deviation for continuous variables or as numbers (percentages) for categorical variables. Variables between the two groups were compared using the chi-square test for categorical variables and t-test or the Mann–Whitney U-test for continuous variables. A paired t-test was used to assess the changes in the continuous variables. Linear regression analysis was used to assess the association between two continuous variables. Further, multiple linear regression analysis was used to evaluate the association of variables with the rate of disease progression, and covariates were selected based on a *p*-value of < 0.2 in the univariate analyses. All *p*-values were two-sided, and a *p*-value < 0.05 was considered significant. SPSS software (Version 26.0; IBM Corporation, Armonk, NY, USA) was used for statistical analysis.

## Results

### Comparison of serum uric acid levels between patients and healthy controls

**Figure 1.**
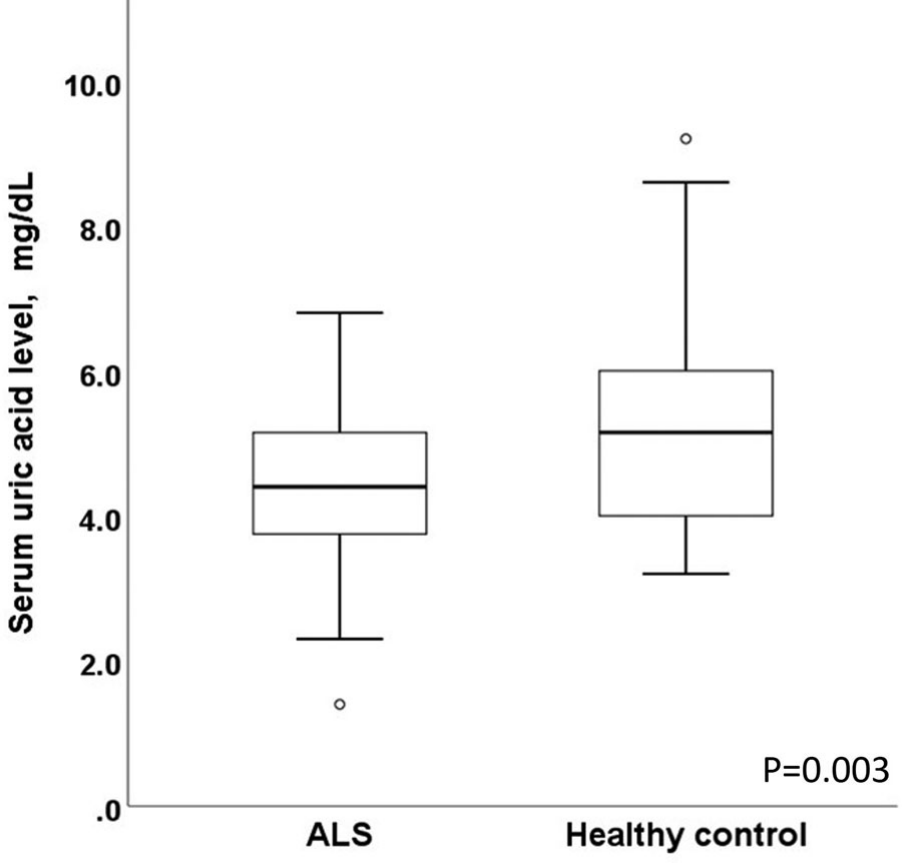
Serum uric acid levels in patients with amyotrophic lateral sclerosis and matched healthy controls. The mean uric acid level in patients with ALS was significantly lower than that of the healthy controls (*p* = 0.003, two-sample t-test).

This study finally included 40 patients (24 men and 16 women). We first compared the serum uric acid levels between patients with ALS and age-, sex-, and BMI-matched healthy controls (Figure 1). The mean uric acid level in patients with ALS (4.5 ± 1.2 mg/dL) was significantly lower than that of the healthy controls (5.3 ± 1.2 mg/dL, *p* = 0.003). In a subgroup analysis by sex, the serum uric acid level was significantly lower in male patients with ALS (4.9 ± 1.2 mg/dL) than in male healthy controls (6.0 ± 1.3 mg/dL, *p* = 0.003). In contrast, although the serum uric acid level in female patients with ALS (3.9 ± 1.1 mg/dL) was lower than that in female healthy controls (4.7 ± 0.6 mg/dL), the difference was not significant (*p* = 0.07).

### Baseline demographics and clinical features

The baseline demographic and clinical features of the included patients are shown in Table 1. The mean age of the patients at the point of initial edaravone treatment was 63 ± 9 years, and the mean disease duration was 14.4 ± 12.2 months. Initially, 28 (70%) and 12 (30%) patients presented with limb weakness and bulbar symptoms, respectively. The mean BMI of the patients was 21.3 ± 3.0 kg/m^2^. Eighteen patients were additionally treated with riluzole, whereas 22 were not. There was no significant association between the baseline uric acid levels and baseline ALSFRS-R scores (*p* = 0.318; Supplementary Figure S1).

**Table 1.** Baseline clinical features of the patients with amyotrophic lateral sclerosis.

Clinical features	Values (n = 40)
Age, years	63 ± 9
Sex, male	24 (60.0)
Disease duration before treatment, months	14.4 ± 12.2
Presenting symptoms	
Limb-onset	28 (70.0)
Bulbar-onset	12 (30.0)
Respiratory-onset	0 (0.0)
Baseline ALSFRS-R^a^ score	34.3 ± 8.3
Baseline Japanese ALS^b^ severity classification	
1	2 (5.0)
2	9 (22.5)
3	7 (17.5)
4	15 (37.5)
5	7 (17.5)
Baseline uric acid level, mg/dL	4.5 ± 1.2

^a^Amyotrophic lateral sclerosis functional rating scale-revised.

^b^Amyotrophic lateral sclerosis.

### Changes in the serum uric acid levels and amyotrophic lateral sclerosis functional rating scale-revised scores

**Figure 2.**
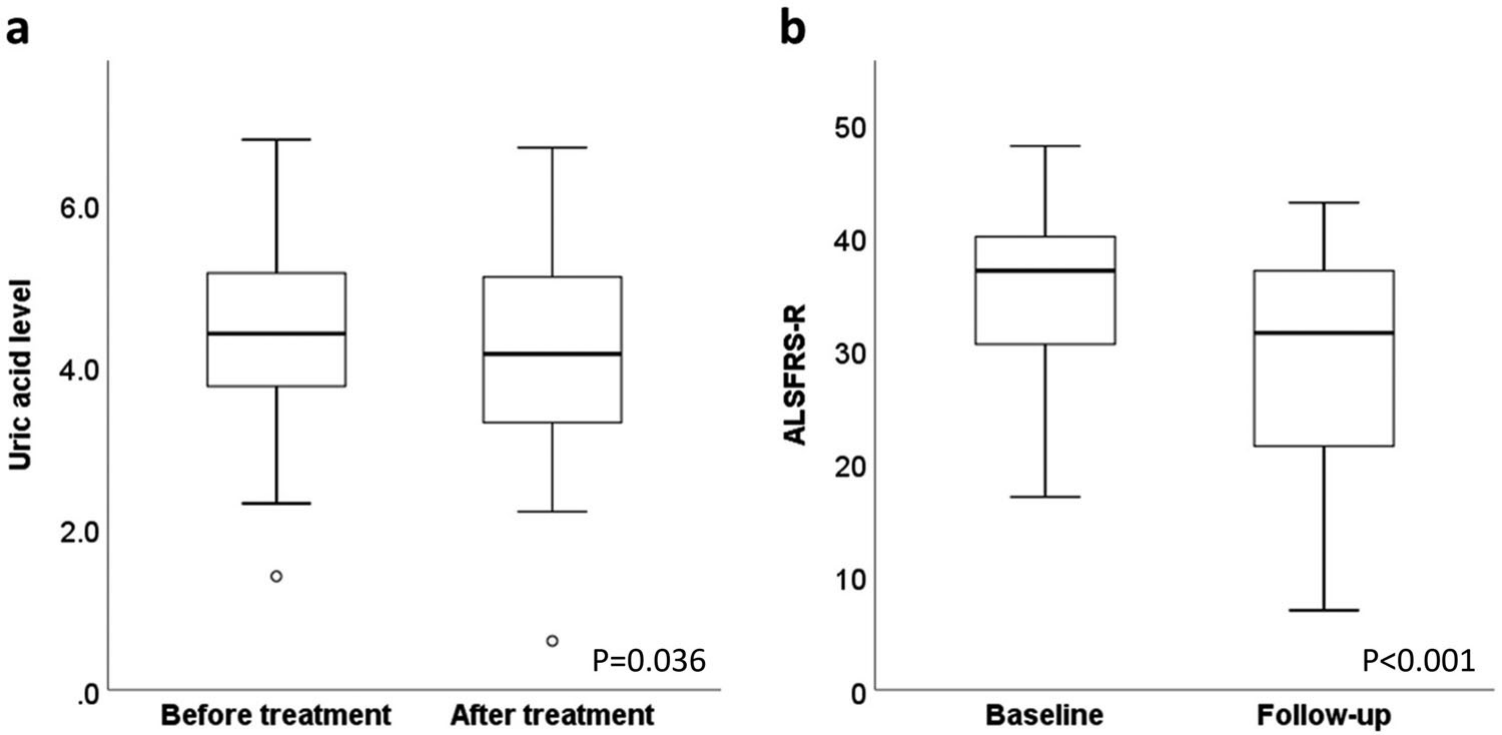
Changes in the (a) uric acid levels before and after edaravone treatment and (b) amyotrophic lateral sclerosis functional rating scale-revised (ALSFRS-R) scores at baseline and follow-up after edaravone treatment. The mean serum uric acid levels significantly decreased after treatment (*p* = 0.036, paired sample t-test). The mean follow-up ALSFRS-R score was lower than the mean baseline ALSFRS-R score (*p* < 0.001, paired sample t-test).

Figure 2a shows the change in serum uric acid levels before and after treatment with edaravone. The mean pre-treatment uric acid level, measured 1.2 ± 1.5 days before the treatment, was 4.5 ± 1.2 mg/dL. The mean post-treatment uric acid level, measured 5.4 ± 2.5 days after the treatment, was 4.2 ± 1.3 mg/dL. The serum uric acid levels decreased after treatment in 26 (65%) patients and increased in 12 (30%) patients. Overall, there was a significant decrease in the serum uric acid levels after treatment with edaravone (*p* = 0.036). We further analyzed the change of uric acid level at different time points and clinical settings. Decrease in serum uric acid level was similarly observed when the pre-treatment uric acid level was compared to the post-treatment serum uric acid levels measured 12 ± 2.9 days after the edaravone administration (4.1 ± 1.3 mg/dL, *p* = 0.016). Among 19 patients whose uric acid levels were measured during the second cycle of edaravone treatment in outpatient setting, significant decrease in serum uric acid levels from 4.4 ± 1.1 mg/dL before the treatment to 4.0 ± 1.1 mg/dL after the treatment was observed (*p* = 0.005).

The average baseline ALSFRS-R score assessed immediately before edaravone treatment was 34.3 ± 8.3, whereas the average follow-up score measured 11.7 ± 5.2 weeks after edaravone treatment was 29.1 ± 10.2 (Figure 2b). The mean rate of change of the ALSFRS-R score was 2.0 ± 2.5 points/month. We further compared the ΔALSFRS-R/month between the 26 patients whose uric acid levels decreased after treatment and the 12 patients whose uric acid levels increased after treatment. The ΔALSFRS-R/month was significantly faster in patients whose uric acid decreased (median 1.5 [Q1 – Q3, 0.7–3.1]) than in patients whose uric acid increased after treatment (0.2 [0–1.0], *p* = 0.021).

### Association between serum uric acid level and progression of amyotrophic lateral sclerosis

**Figure 3.**
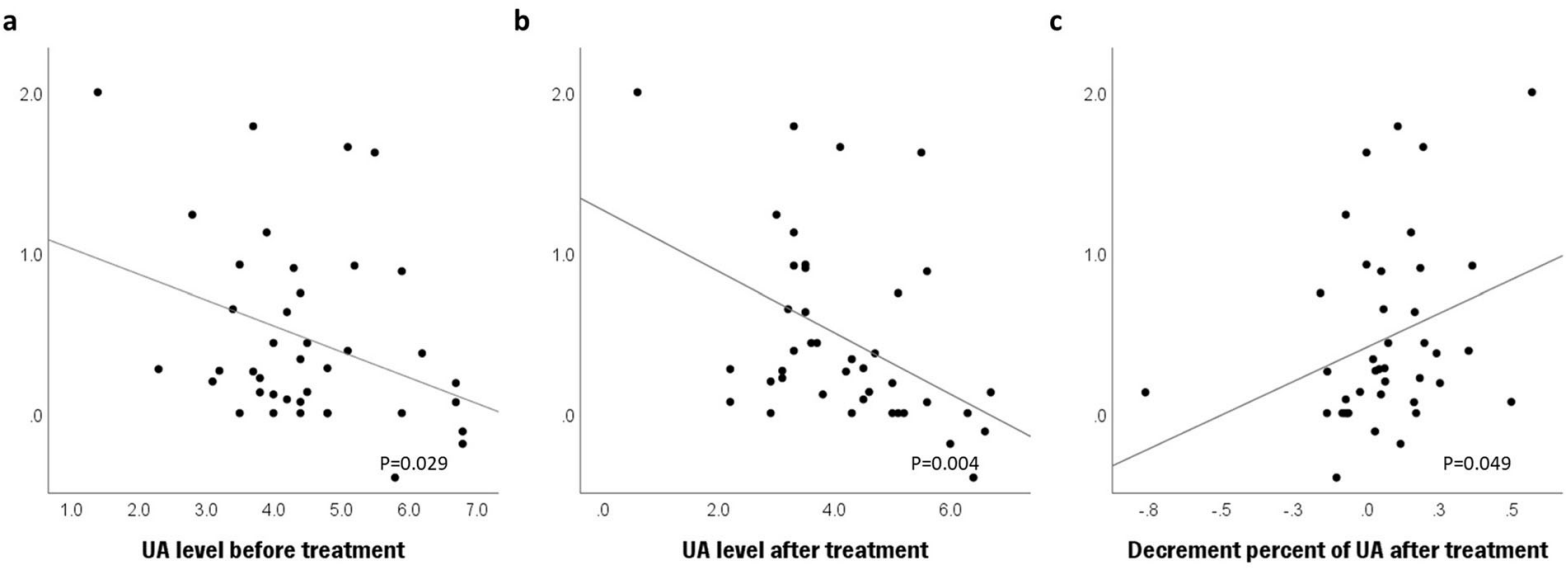
Association between the rate of change of amyotrophic lateral sclerosis functional rating scale-revised (ALSFRS-R) score and (a) pre-treatment uric acid levels, (b) post-treatment uric acid levels, and (c) rate of decline in uric acid after treatment with edaravone. The pre- and post-treatment uric acid levels demonstrated a significant negative correlation with ΔALSFRS-R/month (*p* = 0.024 and *p* = 0.003, univariate linear regression). There was a significant positive correlation between the decrement in uric acid and ΔALSFRS-R/month (*p* = 0.042, univariate linear regression).

The association between the serum uric acid level and degree of disease progression, represented as ΔALSFRS-R/month, is shown in Figure 3. The pre- and post-treatment uric acid levels demonstrated a significant negative correlation with ΔALSFRS-R/month (*p* = 0.024 and *p* = 0.003, respectively), indicating that higher uric acid levels are associated with slower disease progression. In addition, there was a significant positive correlation between the decrement in uric acid and ΔALSFRS-R/month (*p* = 0.042), indicating that a greater rate of decline of uric acid level was associated with rapid disease progression.

### Multivariate analysis to assess clinical factors associated with disease progression

We conducted a multivariate analysis to assess whether uric acid levels were independently associated with disease progression (Table 2). Variables with *p*-value < 0.2 in each univariate analysis were selected into the multivariate analysis. After adjusting for age, initial presenting symptoms, and riluzole administration, the pre-treatment uric acid levels (β = -0.665, *p* = 0.030) and rate of decline in uric acid after treatment (β = 3.573, *p* = 0.041) demonstrated a significant correlation with ΔALSFRS-R/month. The same result was observed when the post-treatment uric acid levels were assessed instead of the pre-treatment uric acid levels and the rate of decline in uric acid after treatment. The post-treatment uric acid level was significantly associated with ΔALSFRS-R/month after adjusting for age, initial presenting symptoms, and riluzole administration (β = −0.758, *p* = 0.009).

**Table 2.** Clinical factors associated with the rate of change of the amyotrophic lateral sclerosis functional rating scale-revised (ALSFRS-R) by multiple linear regression analysis.

Variables	β	*p*-value^a^
Age, years	0.025	0.544
Initial presentation (limb as reference)	1.145	0.143
Riluzole administration (not treated as reference)	1.121	0.129
Pre-treatment uric acid level	−0.665	0.030*
Rate of decline in uric acid after treatment	3.573	0.041*

^a^Multiple linear regression with the rate of change of the amyotrophic lateral sclerosis functional rating scale-revised as the dependent variable.

*p < 0.05.

## Discussion

The current study demonstrates that the serum uric acid level could be a predictor of disease progression in patients with ALS who undergo treatment with edaravone. Serum uric acid levels in patients with ALS were lower than those in healthy controls. Although uric acid levels tended to decrease after treatment with edaravone, uric acid level increased after the treatment in approximately one third of the patients. The rate of decline of ALSFRS-R was faster in patients whose uric acid decreased than in patients whose uric acid increased after treatment. A high baseline uric acid level and low rate of decline in uric acid level after treatment was associated with slower disease progression. This association was still significant after adjusting for age, initial presenting symptoms of ALS, and treatment with riluzole.

Oxidative stress has been suggested to play an important role in the pathogenesis of ALS. Biomarkers of oxidative stress increased in the cerebrospinal fluid of patients with ALS [16–18]. A recent study also provided evidence of increased oxidative stress in the motor cortex of patients with ALS [19]. In contrast, the levels of antioxidant defense biomarkers are decreased in patients with ALS [20, 21]. Uric acid acts as a reactive oxygen species scavenger. Thus, increased oxidative stress in ALS may decrease serum uric acid levels. In fact, previous studies demonstrated that uric acid levels in patients with ALS were significantly lower than that in healthy controls [4, 22]. Conversely, elevated serum uric acid levels may have a protective effect in patients with ALS [5, 7]. Previous reports have shown that in patients with ALS, elevated serum uric acid levels are associated with a lower mortality risk [7, 8, 22], and a high baseline uric acid level is associated with slow disease progression [6, 7].

Edaravone is thought to have antioxidant properties by scavenging hydroxyl radicals and lipid peroxide. It has been shown that the level of 3-nitrotyrosine, a biomarker for oxidative stress, in the cerebrospinal fluid of patients with ALS markedly decreased after treatment with edaravone [23]. Thus, it can be assumed that edaravone and uric acid have synergic effect in scavenging free radicals. This effect was observed in a previous study by Nagase et al. who showed that plasma uric acid levels increased after treatment with edaravone. This effect was especially pronounced among patients who had a satisfactory treatment response in terms of change in the ALSFRS-R score [24]. Based on these findings, we hypothesized that the antioxidant effect of edaravone would be greater in patients whose serum uric acid levels increased after the treatment than in those whose uric acid levels decreased.

Serum uric acid levels tended to decrease after treatment with edaravone. This trend was similarly observed when serum uric acid levels measured at the later phase of the edaravone administration were compared with the baseline values, as well as during the second cycle of edaravone treatment which was conducted in an outpatient setting. It is known that uric acid levels tend to decrease as disease progresses in patient with ALS [5]. Decrease in skeletal muscle mass accompanied by disease progression may also accelerate the decline on uric acid level [25]. Thus, the baseline decline on uric acid level is anticipated in patients with ALS. However, Nagase et al. showed that uric acid levels increased in 10 out of 12 patients after treatment with edaravone [24]. Although not as much as in the previous study, uric acid level increased in 30% of the patients treated with edaravone in the present study, and the rate of disease progression was slower in these patients compared to the patients whose uric acid level decreased. These may suggest the effect of edaravone as a free radical scavenger. However, the proportion of the patients whose uric acid increased after the treatment was relatively smaller than that of the previous study. The discrepancy could be attributed to several factors. First, the sample size was 12 in the previous study and 40 in the current study, both of which are relatively small. Second, the mean baseline ALSFRS-R score in the previous study (40.1 ± 1.2) was higher than that in the current study (34.3 ± 8.3). It is known that edaravone is effective in patients with mild disability [26], and the effect of edaravone could have been less pronounced in the present study.

The baseline uric acid levels and the changes after treatment with edaravone were significantly associated with the rate of change of the ALSFRS-R score. The association between baseline uric acid levels and functional outcomes in patients with ALS has been reported in previous studies. A recent meta-analysis showed that increased uric acid levels were associated with a lower mortality risk in patients with ALS [22]. Keizman et al. demonstrated that the decreased uric acid level in patients with ALS correlated with a decline in the ALSFRS-R score [4]. Similarly, Oh et al. showed that the uric acid level inversely correlated with the rate of change of the ALSFRS-R score [7]. The current study demonstrates that this association is consistently observed among patients treated with edaravone. In addition, changes in the uric acid levels after the administration of edaravone were also associated with the decline in ALSFRS-R scores.

In the current study, the mean rate of change of the ALSFRS-R score was relatively faster than that in previous studies. The mean rate of change of ALSFRS-R was 2.0 ± 2.5 points/month. The rate of change of the ALSFRS-R score was 0.8 points/month in the previous phase-3 trial for edaravone [26] and 0.7 points/month in a long-term follow-up study [27]. The relatively rapid disease progression in this study could be explained by the following aspects. First, the current study was based on a small number of participants, and the result could be significantly influenced by a few patients who experienced severe exacerbation during the study period. In fact, the median (Q1 – Q3) rate of change of ALSFRS-R was 1.2 (0.3–3.6), which was comparable to that of the previous phase-3 trial. Second, patients with rapid disease progression constituted a larger proportion in the current study than in the previous studies. It is known that patients with ALS tend to show a relatively constant disease progression rate, which is maintained throughout the disease course [28]. In a previous phase-3 trial, the duration of the disease and baseline ALSFRS-R score was 1.13 years and 41.9, respectively [26]. In the current study, the patients had a disease duration of 1.2 years with a baseline ALSFRS-R score of 34.3. Thus, the rapid disease progression in the current study may have resulted from patient selection.

The results of the current study corroborate those of previous studies in other aspects. Serum uric acid levels in patients with ALS were significantly lower than those in healthy controls [4, 5, 7, 22]. Similarly, serum uric acid levels were found to be lower in women than in men [3, 4, 6]. There was no significant association between the baseline uric acid levels and baseline ALSFRS-R scores. A previous study also demonstrated the absence of a significant association between uric acid levels and ALSFRS-R scores at a cross-sectional time point [4].

This study had several limitations. First, the study was conducted retrospectively; hence, the monitoring schedule of uric acid levels before and after edaravone administration was inconsistent between patients. Likewise, follow-up ALSFRS-R scores were not assessed at the same time point but at a variable time between 6 and 24 weeks. Second, the association between uric acid levels and long-term outcomes could not be assessed in this study. However, it should be considered that there are some practical difficulties in maintaining edaravone treatment for a long period owing to its high expense and progressive loss of functionality. Third, uric acid levels are influenced by diverse factor including diet, BMI, bone mineral density, and disease severity [2, 21, 29, 30], and these factors were not fully controlled in the present study. Fourth, it cannot be clearly determined whether the change of uric acid level and ALSFRS-R are related to edaravone or not. The patients with slowly progressive ALS may simply had a smaller drop in uric acid independent form edaravone treatment. However, the present study still provides an evidence that uric acid levels are associated with the rate of disease progression in patients with ALS treated with edaravone.

In conclusion, the present study suggests that high baseline serum uric acid levels and low rate of decline in uric acid levels after treatment could be predictors of slow disease progression in patients with ALS. The results of this study may help in the early prediction of the effectiveness of edaravone treatment in ALS patients. Changes in the serum uric acid levels after the first cycle of edaravone treatment could be considered in determining the treatment directions further in patients with ALS.

## Supplementary Material

Supplemental Material
