# Supplementary figures and images for: Serum uric acid level predicts the progression of amyotrophic lateral sclerosis following treatment with edaravone
Source: Redox Rep. 2022 Mar 16;27(1):79–84. doi: 10.1080/13510002.2022.2051964 (PMC8933037; doi:10.1080/13510002.2022.2051964)

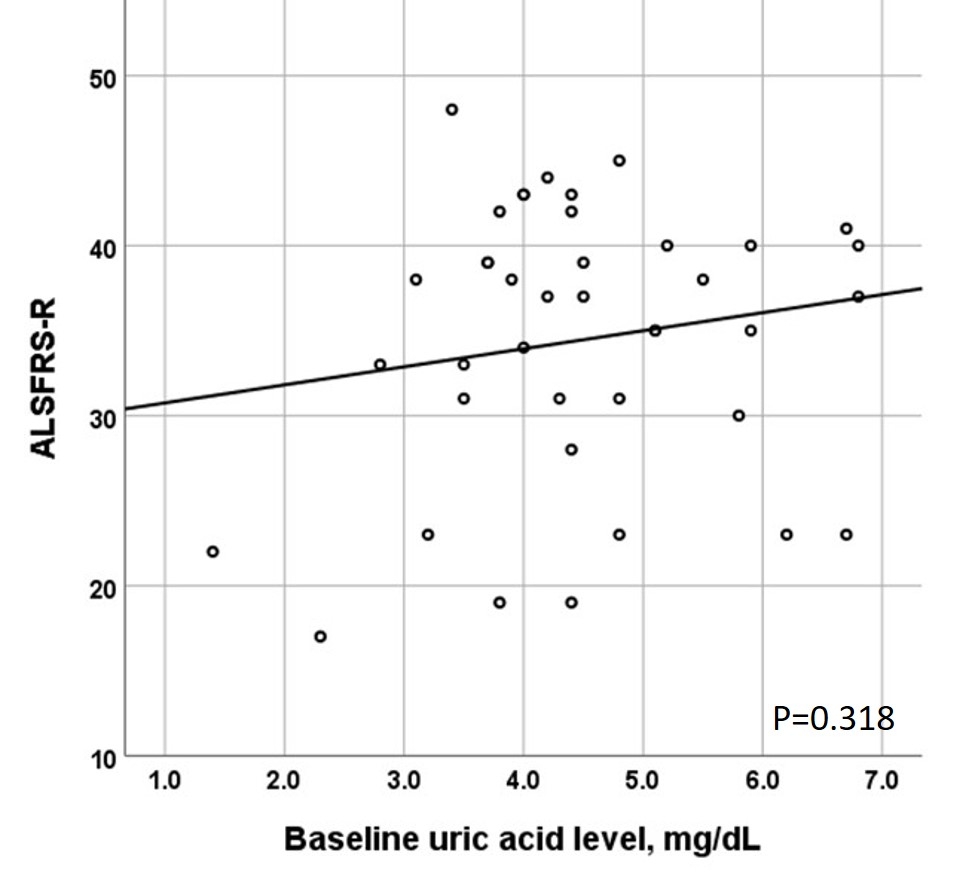

Supplement: Supplemental Material [file YRER_A_2051964_SM2340.jpg]
